# Obituary

**Published:** 1879-08

**Authors:** Abner Hard

**Affiliations:** Aurora, Ill.


### Obituary.

IN MEMORIAM.—Theodore W. Stull, M.D., died at his home in Marengo, McHenry county, Illinois, of phthisis pulmonalis, May 8th, 1879, after a protracted illness. Dr. Stull was born in Smithport, Pennsylvania, in 1833. When six years of age his parents removed to Marengo, Illinois. He was reared upon a farm, received a common English education and taught school for a couple of years, after which he studied medicine, and was graduated at Rush Medical College, Chicago, Ill., Feb. 25th, 1861 He immediately sought a location for the practice of his profession in Missouri, where he remained but a few months. Upon the breaking out of the war, he returned to Illinois, and together with many of his young associates enlisted as a private in Company H. of the Eighth Illinois cavalry, which regiment was being raised at St. Charles, Ill., by Gen. Farnsworth. He was mustered into the U. S. service Sep. 18th, 1861. After the regiment reached Washington, Dr. Stull was appointed hospital steward, and July 1st, 1863, was promoted to assistant surgeon, in which capacity he served until mustered out at the close of the war. The regiment to which he belonged served with distinction in the Army of the Potomac, being engaged in most of the great battles fought by that army, and in many minor engagements in which the cavalry only took part. Both as soldier and surgeon Dr. Stull displayed a noble patriotism, a tender heart and a skill which reflected credit upon his Alma Mater. None but those who have served in a similar capacity can appreciate the responsibility resting upon a surgeon at the time of and after a great battle, or even a small engagement. And it is then that the man, as well as the skillful surgeon, can display his most brilliant qualities. In such positions Dr. Stull was cool, collected and thoughtful, and won the esteem of his fellow-surgeons as well as comrades. But in the desolation which epidemic disease caused in the camp during the long, weary winters, when typhoid fever, dysentery, diarrhœa, measles and small-pox threatened the destruction of our army, the endurance and skill of the surgeons were put to the most trying test. And as one who knows by his presence and participation in these labors, I must say that in camp and hospital Dr. Stull displayed his true worth, and none who received his kind and skillful attention and who lived to recover, but cheerfully give him credit for more than ordinary ability and untiring attention to his patients.

Being of a modest and retiring nature, his abilities were best known to his most intimate friends. At the close of the war he returned to his home in Marengo, Ill., and at once entered into a large and lucrative practice, which he successfully prosecuted until prevented from further labor by the disease which terminated his life. He was a member of the Fox River Valley Medical Association, and contributed much to the usefulness and prosperity of the society. April 15th, 1868, he was married to Miss Aurora M. Adams, of Marengo, Ill. He proved as devoted to his family and domestic life as to his profession. He leaves a wife and three daughters to mourn his early death. Thus are fast passing away the heroes of the late war; but their memories will be cherished by those who knew and could appreciate their worth while memory lasts, or the records of the late war are permitted to endure.

ABNER HARD.

AURORA, ILL., June 10th, 1879.

---

WE regret that in making up the July number an error was made, to the extent of leaving out sixteen pages; that is, from page 97 to page 112 inclusive. We will gladly rectify the error, if subscribers will return the imperfect copies.

We also regret that in some copies of the same number the lithograph illustrating Dr. Earle's article is inverted. This can be corrected when the volume is bound.

